# Differential Predictors of Early and Mid-to-Long Term Cerebrovascular Events Following Transcatheter Aortic Valve Implantation

**DOI:** 10.3390/medicina62091624

**Published:** 2026-08-24

**Authors:** Zeynep Seyma Turinay Ertop, Engin Bozkurt

**Affiliations:** Department of Cardiology, Medicana International Ankara Hospital, Çankaya 06520, Ankara, Turkey; drebozkurt@yahoo.com

**Keywords:** transcatheter aortic valve implantation, TAVI, stroke, cerebrovascular events, post-dilatation, mitral regurgitation, very severe aortic stenosis

## Abstract

*Background and Objectives*: Cerebrovascular events remain among the most feared complications after transcatheter aortic valve implantation (TAVI). Whether early and post-discharge cerebrovascular events are associated with distinct clinical, echocardiographic, haemodynamic, and procedural predictors remains incompletely clarified. *Materials and Methods*: This retrospective, observational, two-center cohort study included 556 patients who underwent TAVI for severe symptomatic aortic stenosis between October 2011 and May 2023. The primary outcomes were in-hospital stroke and mid-to-long term stroke occurring after hospital discharge. Stroke diagnoses were established by neurological assessment and confirmed by cranial computed tomography and/or magnetic resonance imaging. Univariable comparisons and Firth penalized multivariable logistic regression analyses were performed to identify predictors of each outcome. *Results*: In-hospital stroke occurred in 14 patients (2.5%), whereas mid-to-long term stroke occurred in 16 patients (2.9%). In Firth penalized multivariable analysis, baseline mitral regurgitation grade (odds ratio [OR] 2.174, 95% confidence interval [CI] 1.299–3.638; *p* = 0.003), HbA1c (OR 1.644, 95% CI 1.111–2.433; *p* = 0.013), and prior cerebrovascular event (OR 4.269, 95% CI 1.150–15.846; *p* = 0.030) were independently associated with in-hospital stroke. For mid-to-long term stroke, post-dilatation (OR 9.862, 95% CI 2.555–38.067; *p* < 0.001) and very severe aortic stenosis phenotype (OR 3.649, 95% CI 1.343–9.916; *p* = 0.011) were independently associated with the outcome. *Conclusions*: Early and post-discharge cerebrovascular events after TAVI were infrequent but associated with distinct predictor profiles. In-hospital stroke was associated with baseline mitral regurgitation grade, HbA1c, and prior cerebrovascular event, whereas mid-to-long term stroke was associated with post-dilatation and very severe aortic stenosis phenotype. Given the limited number of events, these findings should be interpreted as exploratory and require validation in larger prospective cohorts.

## 1. Introduction

Transcatheter aortic valve implantation (TAVI) has become an established treatment option for patients with severe symptomatic aortic stenosis, with contemporary guidelines supporting its use across an expanding spectrum of surgical risk [1,2]. Landmark randomized trials have demonstrated the efficacy and safety of TAVI in high-, intermediate-, and low-risk populations, supporting its expanding role in contemporary valvular heart disease practice [3,4,5,6]. Parallel improvements in device technology, procedural practice, and accumulated operator experience have been accompanied by better procedural safety and clinical outcomes in contemporary TAVI practice [7,8]. Nevertheless, cerebrovascular events remain among the most feared complications of TAVI because even clinically infrequent events may result in disabling neurological injury, prolonged hospitalization, increased resource utilization, and excess short- and long-term mortality [9,10,11,12,13].

The reported incidence of clinically overt stroke after TAVI varies across trials and registries, but contemporary real-world data generally indicate an early stroke rate of approximately 2–4%, with some administrative datasets reporting slightly higher rates [9,10,11,12,13,14]. Although stroke rates have declined in some registry and trial-level analyses with newer-generation devices and procedural refinements, clinically apparent stroke has not been eliminated [7,8,12].

Importantly, the timing of cerebrovascular events after TAVI may be clinically relevant, as early and post-discharge events may be associated with different risk profiles [11,15]. Distinguishing these temporal patterns may therefore improve the characterization of cerebrovascular risk after TAVI.

Previous studies have identified several clinical and procedural predictors of stroke after TAVI, including age, female sex, prior cerebrovascular disease, atrial fibrillation, peripheral arterial disease, renal dysfunction, non-transfemoral access, valve type, post-dilatation, antithrombotic strategy, and vascular or metabolic risk markers [9,10,14,16,17,18]. However, most available studies have focused on in-hospital or 30-day stroke, whereas fewer have examined later cerebrovascular events [11,15]. Moreover, limited data are available regarding whether early and mid-to-long term cerebrovascular events are associated with distinct baseline echocardiographic, haemodynamic, clinical, and procedural profiles within the same TAVI cohort.

Therefore, the present study aimed to evaluate the incidence and differential predictors of in-hospital and post-discharge mid-to-long term cerebrovascular events in patients undergoing TAVI. We hypothesized that in-hospital and post-discharge cerebrovascular events after TAVI are associated with different clinical, echocardiographic, haemodynamic, and procedural predictors, reflecting partially distinct mechanisms of neurological risk after valve implantation.

## 2. Materials and Methods

### 2.1. Study Design and Population

This was a retrospective, observational, two-center cohort study including all consecutive patients who underwent transcatheter aortic valve implantation for severe symptomatic aortic stenosis between October 2011 and May 2023 at Ataturk Education and Research Hospital and Medicana International Ankara Hospital, Ankara, Turkey. During the study period, a total of 556 patients underwent TAVI at the two participating centers, all of whom were included in the present study. No patients undergoing TAVI during the study period were excluded from the study cohort. The inclusion criteria were TAVI for severe symptomatic aortic stenosis at either participating center during the predefined study period. There were no additional study-specific exclusion criteria.

Severe aortic stenosis was diagnosed according to contemporary guideline-based echocardiographic criteria and confirmed by the institutional heart team [1,2]. The decision to perform TAVI was made by a multidisciplinary heart team based on surgical risk, anatomical suitability, comorbidity burden, frailty, and patient preference. Surgical risk was assessed using the Society of Thoracic Surgeons Predicted Risk of Mortality (STS-PROM) score and categorized as low risk (<4%), intermediate risk (4–8%), or high risk (>8%). Baseline demographic characteristics, cardiovascular comorbidities, functional status, laboratory parameters, echocardiographic measurements, cardiac computed tomography findings, antithrombotic therapy, procedural characteristics, in-hospital complications, and follow-up outcomes were retrospectively collected from institutional electronic medical records and procedural databases.

The study protocol was approved by the local ethics committee of Ataturk Education and Research Hospital (approval no. 2011-68; 18 March 2011). The study was conducted in accordance with the principles of the Declaration of Helsinki. Written informed consent for the TAVI procedure was obtained from all patients before the intervention. No study-specific informed consent was obtained for the present retrospective analysis.

### 2.2. Echocardiographic and Computed Tomography Assessment

All patients underwent transthoracic echocardiographic evaluation before TAVI. Baseline echocardiographic parameters included left ventricular ejection fraction, left atrial diameter, aortic valve area, indexed aortic valve area, peak aortic jet velocity, peak and mean transvalvular gradients, systolic pulmonary artery pressure, mitral regurgitation grade, mitral stenosis, aortic regurgitation grade, transthoracic echocardiographic annulus diameter, and E-wave deceleration time.

Aortic stenosis haemodynamic phenotype was classified into mutually exclusive categories according to guideline-based echocardiographic criteria [1,2]. Patients with very severe aortic stenosis were categorized separately and defined by peak aortic jet velocity ≥ 5.0 m/s and/or mean transvalvular gradient ≥ 60 mmHg. The remaining patients were classified as high-gradient, classical low-flow low-gradient, or paradoxical low-flow low-gradient aortic stenosis according to aortic valve area, transvalvular gradient, left ventricular ejection fraction, and flow status.

Post-procedural echocardiographic evaluation included left ventricular ejection fraction, peak and mean transvalvular gradients, and paravalvular aortic regurgitation grade.

Pre-procedural cardiac computed tomography was used for anatomical assessment and procedural planning. Computed tomography parameters included annular diameters, mean annular diameter, annular radius, annular area, annular perimeter, sinus of Valsalva diameters, mean sinus of Valsalva diameter, mean ascending aortic diameter, bicuspid aortic valve morphology, and aortic valve calcium score when available.

### 2.3. TAVI Procedure and Procedural Variables

All TAVI procedures were performed according to standard institutional practice. The transfemoral access strategy was selected whenever anatomically feasible. Vascular access and closure were performed using percutaneous closure devices or surgical cut-down according to vascular anatomy and operator preference.

Procedural variables included access closure technique, balloon pre-dilatation, post-dilatation, prosthesis size, prosthesis type, valve-in-valve procedure, and device success. Device success was defined according to Valve Academic Research Consortium-3 criteria [19]. Pre-procedural antithrombotic therapy was recorded and classified as aspirin, clopidogrel, warfarin, non-vitamin K antagonist oral anticoagulant, or dual antiplatelet therapy.

### 2.4. Study Outcomes

The two primary outcomes of interest were in-hospital stroke and post-discharge mid-to-long term stroke.

In-hospital stroke was defined as any clinically documented stroke occurring during the index hospitalization after TAVI. Post-discharge mid-to-long term stroke was defined as any clinically documented stroke occurring after hospital discharge during follow-up.

All stroke diagnoses were established by neurological assessment and confirmed by cranial computed tomography and/or magnetic resonance imaging. Stroke events were identified through review of neurological consultation records, neuroimaging reports, discharge summaries, institutional medical records, and follow-up documentation.

In-hospital outcomes included stroke, pericardial tamponade, atrioventricular block, ventricular tachycardia, new-onset atrial fibrillation, left bundle branch block, permanent pacemaker implantation, peripheral vascular complications, other procedural complications, and in-hospital mortality. Follow-up outcomes included 30-day mortality, 6-month mortality, 1-year mortality, post-discharge mid-to-long term stroke, and the last available post-procedural echocardiographic findings.

### 2.5. Statistical Analysis

All statistical analyses were performed using IBM SPSS Statistics, version 25.0 (IBM Corp., Armonk, NY, USA). The distribution of continuous variables was assessed using the Kolmogorov–Smirnov test. Normally distributed continuous variables were expressed as mean ± standard deviation, whereas non-normally distributed variables were expressed as median with interquartile range. Categorical variables were presented as number and percentage. All tests were two-tailed, and a *p*-value of <0.05 was considered statistically significant.

Comparisons between patients with and without in-hospital stroke were performed separately from comparisons between patients with and without post-discharge mid-to-long term stroke. Continuous variables were compared using the independent-samples *t*-test for normally distributed data or the Mann–Whitney U test for non-normally distributed data, as appropriate. Categorical variables were compared using Pearson’s chi-square test or Fisher’s exact test depending on expected cell frequencies.

Univariable analyses were first performed to evaluate associations between candidate clinical, laboratory, echocardiographic, computed tomography, antithrombotic, and procedural variables and each stroke outcome. Variables were selected for multivariable modelling based on clinical relevance and univariable associations. For in-hospital stroke, the candidate variables entered into the multivariable model were age, STS score, prior cerebrovascular event, atrial fibrillation, obstructive coronary artery disease, pre-procedural aspirin use, HbA1c, baseline mitral regurgitation grade, and mitral stenosis. For mid-to-long term stroke, the candidate variables were age, prior cerebrovascular event, atrial fibrillation, post-dilatation, very severe aortic stenosis haemodynamic phenotype, and baseline mean transvalvular gradient.

To identify independent predictors of each outcome, multivariable logistic regression analyses were initially performed using a forward stepwise likelihood ratio method, with an entry threshold of *p* < 0.10 and a removal threshold of *p* > 0.05. Because both stroke outcomes were infrequent and the number of events was limited, Firth penalized logistic regression was additionally applied to reduce small-sample bias and the risk of model instability. The final effect estimates are reported as odds ratios (ORs) with 95% confidence intervals (CIs) derived from the Firth penalized logistic regression models.

Given the limited number of stroke events, multivariable models were deliberately restricted to reduce overfitting, and the findings were interpreted as exploratory and hypothesis-generating rather than confirmatory. Missing data were not imputed; variables with incomplete data were analyzed on an available-case basis. The aortic valve calcium score, available in only 15 patients, was excluded from multivariable modelling on this basis. All other analyzed variables had complete or near-complete data across the 556-patient cohort.

## 3. Results

### 3.1. Baseline Characteristics of the Study Population

A total of 556 patients who underwent TAVI were included in the analysis. The overall cohort comprised predominantly older adults, with a median age of 79.0 years (IQR 73.0–83.0), and 305 patients were female (54.9%). The median body mass index was 27.0 kg/m^2^ (IQR 24.7–29.4). Functional limitation was substantial: 317 patients (57.0%) were in New York Heart Association functional class III, 83 patients (14.9%) were in class IV, and 12 patients (2.2%) presented with acute pulmonary oedema. The median Society of Thoracic Surgeons Predicted Risk of Mortality (STS-PROM) score was 5.4% (IQR 3.7–7.6). According to STS-PROM risk categories, 169 patients (30.4%) were classified as low surgical risk (<4%), 264 (47.5%) as intermediate surgical risk (4–8%), and 123 (22.1%) as high surgical risk (>8%). Baseline demographic characteristics, comorbidities, and laboratory parameters are summarized in Table 1.

The burden of cardiovascular comorbidity was considerable. Hypertension was present in 462 patients (83.1%), hyperlipidaemia in 280 patients (50.4%), chronic obstructive pulmonary disease in 263 patients (47.3%), diabetes mellitus in 164 patients (29.5%), and atrial fibrillation in 132 patients (23.7%). Prior cerebrovascular events were documented in 33 patients (5.9%), and neurological disease was present in 26 patients (4.7%). Obstructive coronary artery disease requiring revascularization was present in 304 patients (54.7%). Among the 70 patients who underwent PCI in relation to TAVI, PCI was performed before TAVI in 53 patients (75.7%), concomitantly with TAVI in 10 patients (14.3%), and after TAVI in 7 patients (10.0%). The median PCI-to-TAVI interval was 7.0 days (IQR 4.0–15.0).

Baseline laboratory assessment showed a median estimated glomerular filtration rate of 65.0 mL/min/1.73 m^2^ (IQR 50.0–81.9) and a median NT-proBNP level of 1822.0 pg/mL (IQR 757.0–5116.0), reflecting the haemodynamic burden and comorbidity profile of the cohort.

### 3.2. Baseline Echocardiographic and Computed Tomography Findings

Baseline echocardiography demonstrated a median left ventricular ejection fraction of 55.0% (IQR 45.0–65.0). The median aortic valve area was 0.7 cm^2^ (IQR 0.6–0.8), and the median indexed aortic valve area was 0.4 cm^2^/m^2^ (IQR 0.3–0.4). The median peak aortic jet velocity was 4.4 m/s (IQR 4.1–4.8), the median peak transvalvular gradient was 78.5 mmHg (IQR 67.3–93.0), and the median mean transvalvular gradient was 46.0 mmHg (IQR 41.0–58.0). Baseline echocardiographic and computed tomography findings are summarized in Table 2.

The predominant haemodynamic phenotype was high-gradient aortic stenosis, present in 380 patients (68.3%), followed by very severe aortic stenosis in 134 patients (24.1%), low-flow low-gradient aortic stenosis in 35 patients (6.3%), and paradoxical low-flow low-gradient aortic stenosis in 7 patients (1.3%). Clinically relevant mitral regurgitation, defined as grade ≥ 2, was present in 186 patients (33.5%). Bicuspid aortic valve morphology was identified in 71 patients (12.8%).

Cardiac computed tomography was used to characterize aortic root and annular anatomy. The median CT-derived mean annulus diameter was 24.5 mm (IQR 23.5–25.5), the median CT annulus area was 471.2 mm^2^ (IQR 433.5–510.4), and the median ascending aortic diameter was 36.0 mm (IQR 34.0–38.5). Aortic valve calcium scoring was available in only 15 patients, with a median Agatston score of 2860 (IQR 1275–5039).

### 3.3. Procedural Characteristics and Clinical Outcomes

Pre-procedural antithrombotic therapy was predominantly aspirin-based, used in 405 patients (72.8%). Warfarin was used in 110 patients (19.8%), non-vitamin K antagonist oral anticoagulants in 19 patients (3.4%), dual antiplatelet therapy in 19 patients (3.4%), and clopidogrel alone in 3 patients (0.5%). Procedural characteristics and antithrombotic therapy are presented in Table 3.

Transfemoral access closure was performed using Proglide in 365 patients (65.6%) and Prostar in 179 patients (32.2%), while surgical cut-down was required in 12 patients (2.2%). Balloon pre-dilatation was performed in 400 patients (71.9%), and post-dilatation was performed in 19 patients (3.4%). The most frequently implanted prosthesis was SAPIEN XT in 479 patients (86.2%), followed by SAPIEN 3 in 46 patients (8.3%) and Lotus in 24 patients (4.3%). Device success according to VARC-3 criteria was achieved in 535 patients (96.2%). Prosthesis types and clinical outcomes are summarized in Table 4.

In-hospital stroke occurred in 14 patients (2.5%). Other in-hospital events included complete atrioventricular block in 38 patients (6.8%), new-onset atrial fibrillation in 20 patients (3.6%), left bundle branch block in 14 patients (2.5%), and permanent pacemaker implantation in 40 patients (7.2%). Peripheral vascular complications occurred in 37 patients (6.7%), and pericardial tamponade occurred in 10 patients (1.8%). In-hospital mortality occurred in 22 patients (4.0%).

Post-procedural echocardiography demonstrated a marked reduction in transvalvular gradients. The median post-procedural peak gradient was 19.0 mmHg (IQR 16.0–23.0), and the median mean gradient was 10.0 mmHg (IQR 8.0–12.0). The median post-procedural left ventricular ejection fraction was 60.0% (IQR 45.8–65.0). Paravalvular aortic regurgitation was mild in 531 patients (95.5%) and moderate in 25 patients (4.5%); no severe paravalvular leak was recorded.

During follow-up, all-cause mortality was 5.9% at 30 days, 7.2% at 6 months, and 16.4% at 1 year. Mid-to-long term stroke, defined as any cerebrovascular event occurring after hospital discharge, was documented in 16 patients (2.9%).

### 3.4. Predictors of In-Hospital Stroke

In-hospital stroke occurred in 14 of 556 patients (2.5%). Patients who experienced in-hospital stroke were numerically older than those without in-hospital stroke, although this difference was not statistically significant. The median age was 84.0 years in patients with in-hospital stroke and 79.0 years in those without in-hospital stroke (*p* = 0.342). The median STS score was also numerically higher among patients with in-hospital stroke, but the difference did not reach statistical significance. Univariable comparisons according to in-hospital stroke status are presented in Table 1, Table 2 and Table 3.

Among demographic and clinical variables, prior cerebrovascular event was more frequent in patients who developed in-hospital stroke than in those who did not. A prior cerebrovascular event was present in 3 of 14 patients with in-hospital stroke (21.4%) and in 30 of 542 patients without in-hospital stroke (5.5%; *p* = 0.044). Obstructive coronary artery disease was numerically more common among patients with in-hospital stroke, but this association did not reach statistical significance.

Baseline echocardiographic and computed tomography comparisons showed that mitral regurgitation grade differed significantly according to in-hospital stroke status (*p* = 0.039). None or trace mitral regurgitation was present in 23.8% of patients without in-hospital stroke but in none of the patients who developed in-hospital stroke. Moderate-to-severe or severe mitral regurgitation was observed in 28.6% of patients with in-hospital stroke compared with 12.0% of patients without in-hospital stroke. Mitral stenosis was more frequent in the in-hospital stroke group, although this association was of borderline statistical significance. No significant differences were observed for left ventricular ejection fraction, aortic valve area, transvalvular gradients, aortic stenosis haemodynamic phenotype, or CT-derived annular and supra-annular measurements.

Procedural characteristics and pre-procedural antithrombotic therapy were generally comparable between patients with and without in-hospital stroke. No statistically significant differences were observed in antithrombotic regimen, transfemoral closure technique, balloon pre-dilatation, post-dilatation, prosthesis size, valve-in-valve procedure, or device success.

In Firth penalized multivariable logistic regression analysis (Table 5), baseline mitral regurgitation grade, HbA1c, and prior cerebrovascular event were independently associated with in-hospital stroke. Baseline mitral regurgitation grade was associated with increased odds of in-hospital stroke (OR 2.174, 95% CI 1.299–3.638; *p* = 0.003), as was HbA1c (OR 1.644, 95% CI 1.111–2.433; *p* = 0.013). Prior cerebrovascular event was also independently associated with in-hospital stroke (OR 4.269, 95% CI 1.150–15.846; *p* = 0.030). Given the limited number of in-hospital stroke events, these findings should be interpreted as exploratory and hypothesis-generating.

### 3.5. Predictors of Mid-to-Long Term Stroke

Mid-to-long term stroke occurred in 16 of 556 patients (2.9%) after hospital discharge. Baseline demographic characteristics and comorbidities were broadly similar between patients with and without mid-to-long term stroke. Median age, sex distribution, body mass index, NYHA functional class, STS score, diabetes mellitus, hypertension, hyperlipidaemia, atrial fibrillation, prior cerebrovascular events, chronic obstructive pulmonary disease, renal function, HbA1c, lipid profile, platelet count, and NT-proBNP did not differ significantly between groups. Univariable comparisons according to mid-to-long term stroke status are presented in Table 1, Table 2 and Table 3.

Among echocardiographic parameters, left ventricular ejection fraction and peak and mean transvalvular gradients did not differ significantly between patients with and without mid-to-long term stroke. Very severe aortic stenosis was present in 8 of 16 patients with mid-to-long term stroke (50.0%) and in 126 of 540 patients without mid-to-long term stroke (23.3%); however, this difference did not reach statistical significance in the univariable analysis. CT-derived anatomical parameters, mitral regurgitation grade, aortic regurgitation grade, mitral stenosis, and bicuspid aortic valve morphology were comparable between groups.

Procedural and antithrombotic comparisons showed no significant differences in pre-procedural antithrombotic regimen, transfemoral closure technique, balloon pre-dilatation, prosthesis size, valve-in-valve procedure, or device success. However, post-dilatation was performed significantly more often in patients who subsequently developed mid-to-long term stroke than in those who did not. Post-dilatation was used in 3 of 16 patients with mid-to-long term stroke (18.8%) and in 16 of 540 patients without mid-to-long term stroke (3.0%; *p* = 0.014).

In Firth penalized multivariable logistic regression analysis (Table 5), post-dilatation and the very severe aortic stenosis phenotype were independently associated with mid-to-long term stroke. Post-dilatation was associated with increased odds of mid-to-long term stroke (OR 9.862, 95% CI 2.555–38.067; *p* < 0.001), while the very severe aortic stenosis phenotype was also independently associated with the outcome (OR 3.649, 95% CI 1.343–9.916; *p* = 0.011). Given the limited number of outcome events, these findings should be interpreted as exploratory and hypothesis-generating pending validation in larger prospective cohorts.

## 4. Discussion

In this retrospective cohort of 556 patients undergoing TAVI for severe symptomatic aortic stenosis, the incidence of in-hospital stroke was 2.5%, whereas mid-to-long term stroke after hospital discharge occurred in 2.9% of patients. The principal finding of the present study was that early and later cerebrovascular events were associated with different predictor profiles. In-hospital stroke was independently associated with baseline mitral regurgitation grade, HbA1c, and prior cerebrovascular event, while mid-to-long term stroke was independently associated with post-dilatation and very severe aortic stenosis haemodynamic phenotype. These findings support the concept that cerebrovascular risk after TAVI is temporally heterogeneous and may reflect different mechanisms during the index hospitalization and after discharge.

The observed in-hospital stroke rate in our cohort is consistent with contemporary real-world TAVI data. Large registries and observational studies have reported early or in-hospital stroke rates generally ranging from approximately 2% to 4%, with some administrative datasets reporting slightly higher rates, depending on patient population, procedural era, stroke definition, access route, and valve technology [9,10,11,12,13,14]. In the UK TAVI registry, the incidence of in-hospital stroke was 2.4%, closely paralleling the rate observed in our cohort [9]. Similarly, contemporary national registry data from the Netherlands reported an in-hospital stroke rate of 2.0%, whereas the SwissTAVI Registry reported a 30-day stroke incidence of 3.0%, with 69% of events occurring within the first 48 h after TAVI [10,11]. A pooled patient-level analysis of transfemoral TAVI studies also reported a 30-day stroke incidence of 2.2%, highlighting the persistence of clinically relevant neurological risk despite contemporary procedural practice [12]. Taken together, these data emphasize that clinically apparent stroke remains an infrequent but clinically important complication of TAVI.

Most previous studies have focused on early neurological events, particularly in-hospital or 30-day stroke [9,10,14,16,17,18]. Early stroke after TAVI is generally attributed to procedure-related embolic and haemodynamic mechanisms, including catheter and wire manipulation, interaction with the calcified native aortic valve, balloon valvuloplasty, valve deployment, post-dilatation, aortic arch atheroma, rapid pacing-related hypotension, and periprocedural rhythm disturbances [20,21]. However, prediction of early stroke remains challenging because the event is multifactorial and may not be fully explained by a limited number of clinical or procedural variables [18,22]. This is reflected by the heterogeneity of predictors reported across studies, including age, female sex, prior cerebrovascular disease, peripheral arterial disease, non-transfemoral access, valve type, post-dilatation, vascular or metabolic risk markers, and antithrombotic strategy [9,10,14,16,17,18].

In our analysis, baseline mitral regurgitation grade emerged as an independent predictor of in-hospital stroke. This finding should be interpreted cautiously, because mitral regurgitation is unlikely to represent a direct embolic mechanism. Rather, more advanced mitral regurgitation may identify a subgroup with more complex haemodynamic disease, higher left atrial pressure, atrial remodelling, pulmonary congestion, impaired cardiac reserve, or greater vulnerability to periprocedural haemodynamic instability. This interpretation is consistent with the broader concept that neurological risk after TAVI may reflect both patient-related vulnerability and procedure-related factors rather than a single isolated mechanism [23]. Although atrial fibrillation itself was not independently associated with in-hospital stroke in the present cohort, mitral regurgitation may still act as an integrated marker of structural and haemodynamic vulnerability. Therefore, the association between mitral regurgitation and early stroke in our study should be viewed primarily as hypothesis-generating and warrants validation in larger cohorts with detailed rhythm monitoring and left atrial structural assessment.

HbA1c was also independently associated with in-hospital stroke. This finding is biologically plausible, as chronic dysglycaemia is associated with endothelial dysfunction, systemic inflammation, platelet activation, impaired fibrinolysis, accelerated atherosclerosis, and microvascular disease [24,25,26]. These mechanisms may increase susceptibility to clinically overt neurological injury during procedural embolic exposure or transient haemodynamic compromise [20,21,24,25,26]. Previous TAVI studies have also suggested that vascular and metabolic risk markers, including lipid-related parameters and comorbid vascular risk factors, may contribute to stroke risk after TAVI [14,17]. In this context, HbA1c may reflect not only glycaemic control but also a broader prothrombotic and vascular risk phenotype [24,25,26]. Nevertheless, because of the low number of in-hospital stroke events, this association should not be interpreted as proof of causality. Prior cerebrovascular event was also independently associated with in-hospital stroke in the Firth penalized model. This finding is consistent with previous TAVI studies identifying a history of cerebrovascular disease as a marker of increased periprocedural neurological risk [9,10,14,16,17,18]. A prior cerebrovascular event may reflect an underlying burden of cerebrovascular and systemic atherosclerotic disease, potentially increasing vulnerability to embolic or haemodynamic insults during TAVI. However, the confidence interval around this association was wide, reflecting the small number of in-hospital stroke events and the limited number of patients with both a prior cerebrovascular event and an in-hospital stroke. Therefore, this finding should also be interpreted cautiously and requires confirmation in larger cohorts.

The predictors of post-discharge mid-to-long term stroke differed from those of in-hospital stroke. Post-dilatation was strongly associated with post-discharge cerebrovascular events in our cohort. Prior studies have reported post-dilatation as a predictor of early stroke, potentially related to additional mechanical manipulation of the implanted valve and native calcified leaflets [10,14,20,21]. In our cohort, however, post-dilatation was associated with mid-to-long term rather than in-hospital stroke. The mechanism underlying this association cannot be determined from the present data. Post-dilatation may represent a marker of greater anatomical or procedural complexity rather than a direct cause of subsequent cerebrovascular events. Importantly, aortic valve calcium scores were available in only 15 patients, precluding a meaningful analysis of the relationship among calcific burden, post-dilatation, and subsequent stroke. Therefore, any mechanistic interpretation of this association remains speculative and requires confirmation in studies with systematic CT-based calcium quantification.

Very severe aortic stenosis haemodynamic phenotype was also independently associated with post-discharge mid-to-long term stroke. This observation is of potential interest because later stroke after TAVI is generally considered to be influenced predominantly by patient-related thromboembolic and vascular factors, including atrial fibrillation, prior cerebrovascular disease, vascular risk factors, and post-discharge antithrombotic status [11,15,23]. A very severe aortic stenosis phenotype may reflect more advanced pre-procedural haemodynamic and structural disease; however, the mechanisms underlying its association with subsequent cerebrovascular events cannot be determined from the present data. Although baseline haemodynamic factors, including higher mean aortic gradients, have been associated with later cerebrovascular events in some previous cohorts [15], the present finding should be regarded as exploratory and hypothesis-generating. In particular, it should not be interpreted as evidence of a causal relationship between very severe aortic stenosis and post-discharge stroke. Validation in larger prospective cohorts with contemporary TAVI devices and systematic longitudinal assessment is required before this finding can be incorporated into clinical risk stratification.

The distinction between early and later cerebrovascular events has practical implications. Early stroke prevention is primarily focused on procedural optimization, careful device manipulation, access route selection, minimization of unnecessary ballooning, and selective consideration of cerebral embolic protection [20,21]. Although cerebral embolic protection devices can capture embolic debris and may reduce new cerebral lesion burden in some studies, randomized evidence has not demonstrated a significant reduction in the primary endpoint of clinically overt periprocedural stroke with routine cerebral embolic protection [20,21,27]. In contrast, prevention of later cerebrovascular events may require attention to post-discharge factors, including rhythm surveillance, optimization of antithrombotic therapy according to established clinical indications, aggressive vascular risk factor control, and identification of patients with high-risk haemodynamic or procedural profiles [11,15,23]. Our findings suggest the value of a more temporally nuanced approach to stroke risk assessment after TAVI rather than treating all cerebrovascular events as a single homogeneous endpoint.

The present study has several clinical and research implications. First, it reinforces that the incidence of clinically apparent stroke after TAVI in contemporary practice is low but not negligible [9,10,11,12,13,14]. Second, it suggests that in-hospital and post-discharge stroke may have partially distinct predictors, supporting separate analysis of these endpoints in future studies [11,15]. Third, it highlights the potential importance of baseline mitral regurgitation, glycaemic status, prior cerebrovascular history, post-dilatation, and very severe aortic stenosis phenotype as candidate markers for neurological risk assessment. Finally, these findings should be considered exploratory and should prompt further investigation in larger, multicentre cohorts with standardized neurological assessment, systematic neuroimaging, detailed CT-based calcium quantification, procedural imaging data, rhythm monitoring, and longitudinal antithrombotic treatment data.

## 5. Limitations

Several limitations of the present study should be acknowledged. First, this was a retrospective observational study conducted at two tertiary centers, and therefore the findings may not be fully generalizable to other TAVI programs with different patient selection criteria, procedural volumes, valve preferences, imaging protocols, or antithrombotic strategies. Second, the study period extended over more than a decade, during which TAVI technology, procedural planning, operator experience, valve platforms, and post-procedural care evolved. This temporal heterogeneity may have influenced both procedural characteristics and neurological outcomes. In addition, the overall procedural volume was distributed over a prolonged study period, and potential changes in institutional and operator experience over time may have influenced the observed outcomes. Furthermore, the cohort predominantly comprised patients treated with the second-generation balloon-expandable SAPIEN XT valve, which accounted for 86.2% of implanted prostheses. Because contemporary TAVI practice increasingly relies on newer-generation balloon-expandable and self-expanding devices, the applicability of the present findings to current TAVI populations may be limited. Accordingly, these associations require confirmation in contemporary cohorts treated with newer-generation transcatheter heart valves.

Third, the number of cerebrovascular events was low. In-hospital stroke occurred in 14 patients, and mid-to-long term stroke occurred in 16 patients. Although this incidence is consistent with contemporary TAVI literature, the limited number of events restricts statistical power, reduces the number of variables that can be reliably included in multivariable models, and increases the risk of overfitting. Accordingly, the regression findings should be interpreted as exploratory and hypothesis-generating rather than confirmatory.

Fourth, despite adjustment for clinically relevant variables, residual confounding cannot be excluded because of the retrospective design. Data on potentially relevant factors such as detailed neuroimaging characteristics, aortic arch atheroma burden, intra-procedural haemodynamic instability, embolic protection device use, post-procedural rhythm monitoring, and changes in antithrombotic therapy during follow-up were not systematically available. Mitral regurgitation aetiology was also not systematically recorded; therefore, functional and degenerative mitral regurgitation could not be reliably distinguished, limiting further mechanistic interpretation of the observed association between mitral regurgitation grade and in-hospital stroke. Fifth, aortic valve calcium scoring was available in only a small subset of patients, precluding meaningful assessment of the relationship between calcific burden, post-dilatation, and cerebrovascular risk.

Finally, only clinically documented stroke events were evaluated. Although stroke diagnoses were established by neurological assessment and confirmed by cranial computed tomography and/or magnetic resonance imaging, systematic post-procedural brain imaging was not performed in all patients; therefore, silent cerebral ischaemic lesions could not be assessed. In addition, the database did not record the date or timing of individual post-discharge stroke events; only the occurrence of stroke during follow-up was captured. Because event times were unavailable, a valid time-to-event structure could not be defined, precluding Kaplan–Meier stroke-free survival analysis and Fine–Gray competing-risks regression, the latter being particularly relevant given the competing risk of mortality in this cohort. Therefore, the mid-to-long term stroke analysis should be interpreted as an analysis of cumulative occurrence rather than time-dependent risk. Future prospective studies should systematically record the timing of cerebrovascular events to enable time-to-event and competing-risk analyses.

## 6. Conclusions

In this retrospective cohort of patients undergoing TAVI for severe symptomatic aortic stenosis, clinically apparent cerebrovascular events were infrequent but clinically relevant. In-hospital stroke occurred in 2.5% of patients, whereas mid-to-long term stroke after hospital discharge occurred in 2.9%.

The predictors of early and later cerebrovascular events differed. In-hospital stroke was independently associated with baseline mitral regurgitation grade, HbA1c, and prior cerebrovascular event, whereas mid-to-long term stroke was independently associated with post-dilatation and very severe aortic stenosis haemodynamic phenotype. These findings suggest that cerebrovascular risk after TAVI may be temporally heterogeneous and may be associated with distinct early and post-discharge risk profiles.

Because of the limited number of stroke events, these results should be considered hypothesis-generating. Larger prospective multicenter studies with standardized neurological assessment, systematic imaging, detailed procedural data, and longer follow-up are warranted to validate these findings and refine risk stratification for cerebrovascular events after TAVI.

## Figures and Tables

**Table 1:** Baseline Demographic Characteristics, Comorbidities, and Laboratory Parameters—Overall Cohort and by Stroke Status.

Variable	Overall (*n* = 556)	No IH Stroke (*n* = 542)	IH Stroke (*n* = 14)	*p* (IH)	No ML Stroke (*n* = 540)	ML Stroke (*n* = 16)	*p* (ML)
**Demographic Characteristics**							
Age (years)	79.0 (73.0–83.0)	79.0 (73.0–83.0)	84.0 (63.8–91.8)	0.342	79.0 (73.0–83.0)	79.5 (77.0–83.0)	0.515
Female sex, *n* (%)	305 (54.9)	299 (55.2)	6 (42.9)	0.421	297 (55.0)	8 (50.0)	0.692
Body mass index (kg/m^2^)	27.0 (24.7–29.4)	27.0 (24.8–29.4)	24.4 (22.7–30.1)	0.102	27.0 (24.7–29.4)	27.3 (25.2–28.7)	0.870
NYHA functional class, *n* (%)				0.670			0.640
Grade II	144 (25.9)	142 (26.2)	2 (14.3)		141 (26.1)	3 (18.8)	
Grade III	317 (57.0)	307 (56.6)	10 (71.4)		308 (57.0)	9 (56.3)	
Grade IV	83 (14.9)	81 (14.9)	2 (14.3)		79 (14.6)	4 (25.0)	
Acute pulmonary oedema	12 (2.2)	12 (2.2)	0 (0.0)		12 (2.2)	0 (0.0)	
STS score (%)	5.4 (3.7–7.6)	5.4 (3.6–7.5)	6.5 (4.4–8.3)	0.156	5.4 (3.7–7.6)	4.5 (2.8–6.7)	0.238
**Cardiovascular History and Comorbidities**							
Diabetes mellitus, *n* (%)	164 (29.5)	158 (29.2)	6 (42.9)	0.372	160 (29.6)	4 (25.0)	0.788
Hypertension, *n* (%)	462 (83.1)	450 (83.0)	12 (85.7)	>0.999	449 (83.1)	13 (81.3)	0.741
Hyperlipidaemia, *n* (%)	280 (50.4)	270 (49.8)	10 (71.4)	0.110	273 (50.6)	7 (43.8)	0.592
Atrial fibrillation, *n* (%)	132 (23.7)	126 (23.2)	6 (42.9)	0.109	128 (23.7)	4 (25.0)	>0.999
COPD, *n* (%)	263 (47.3)	257 (47.4)	6 (42.9)	0.736	254 (47.0)	9 (56.3)	0.467
Pulmonary arterial hypertension, *n* (%)	43 (7.7)	41 (7.6)	2 (14.3)	0.296	43 (8.0)	0 (0.0)	0.626
Prior cerebrovascular event, *n* (%)	33 (5.9)	30 (5.5)	3 (21.4)	0.044	33 (6.1)	0 (0.0)	0.615
Neurological disease, *n* (%)	26 (4.7)	26 (4.8)	0 (0.0)	>0.999	24 (4.4)	2 (12.5)	0.169
Dialysis-dependent renal failure, *n* (%)	13 (2.3)	13 (2.4)	0 (0.0)	>0.999	13 (2.4)	0 (0.0)	>0.999
Prior valve surgery, *n* (%)	24 (4.3)	24 (4.4)	0 (0.0)	0.654	24 (4.4)	0 (0.0)	>0.999
Prior CABG, *n* (%)	130 (23.4)	126 (23.2)	4 (28.6)	0.749	128 (23.7)	2 (12.5)	0.383
Prior PCI/coronary stenting, *n* (%)	115 (20.7)	111 (20.5)	4 (28.6)	0.502	113 (20.9)	2 (12.5)	0.544
Obstructive CAD (revascularised), *n* (%)	304 (54.7)	293 (54.1)	11 (78.6)	0.069	297 (55.0)	7 (43.8)	0.373
PCI timing, *n* (%) †				>0.999			0.243
Before TAVI	53 (75.7)	50 (74.6)	3 (100.0)		53 (76.8)	0 (0.0)	
Concomitant with TAVI	10 (14.3)	10 (14.9)	0 (0.0)		9 (13.0)	1 (100.0)	
After TAVI	7 (10.0)	7 (10.4)	0 (0.0)		7 (10.1)	0 (0.0)	
PCI-to-TAVI interval (days), median (IQR)	7.0 (4.0–15.0)	7.0 (3.3–15.0)	5.0 (4.0–25.0)	0.884	7.0 (4.0–15.0)	N/A	—
**Laboratory Parameters**							
Baseline eGFR (mL/min/1.73 m^2^)	65.0 (50.0–81.9)	65.6 (50.0–82.0)	56.5 (42.8–79.8)	0.344	65.0 (50.0–82.0)	67.0 (44.5–71.8)	0.502
HbA1c (%)	6.0 (5.6–6.6)	6.0 (5.6–6.6)	6.1 (5.3–8.6)	0.446	6.0 (5.6–6.6)	6.0 (5.8–6.4)	0.982
Low-density lipoprotein cholesterol (mg/dL)	95.5 (73.1–120.9)	95.4 (73.0–120.4)	103.5 (86.2–124.5)	0.459	95.5 (73.1–120.4)	100.6 (71.3–131.8)	0.768
High-density lipoprotein cholesterol (mg/dL)	44.0 (36.1–53.0)	44.0 (36.2–53.0)	44.5 (32.0–49.3)	0.711	44.0 (36.7–53.0)	35.4 (28.1–54.3)	0.183
Platelet count (×10^3^/µL)	228.0 (185.0–283.0)	227.5 (185.0–284.0)	243.5 (202.0–253.8)	0.858	228.0 (185.0–283.8)	223.5 (194.5–262.0)	0.988
Baseline NT-proBNP (pg/mL)	1822 (757–5116)	1759 (757–5139)	3985 (1361–5382)	0.375	1822 (759–5204)	1773 (636–4736)	0.380

Data are presented as median (interquartile range, IQR) for continuous variables, and as number (*n*) and percentage (%) for categorical variables. Between-group comparisons were performed using the Mann–Whitney U test for continuous variables and Fisher’s exact test or the chi-square test for categorical variables. Bold *p*-values denote statistical significance (*p* < 0.05). Abbreviations: IQR, interquartile range; NYHA, New York Heart Association; STS, Society of Thoracic Surgeons; COPD, chronic obstructive pulmonary disease; CABG, coronary artery bypass grafting; PCI, percutaneous coronary intervention; CAD, coronary artery disease; eGFR, estimated glomerular filtration rate; HbA1c, glycated haemoglobin; NT-proBNP, N-terminal pro-B-type natriuretic peptide; IH, in-hospital; ML, mid-to-long term, N/A, not applicable. † Percentages for PCI timing refer to the 70 patients who underwent PCI.

**Table 2:** Baseline Echocardiographic and Computed Tomography Parameters—Overall Cohort and by Stroke Status.

Variable	Overall (*n* = 556)	No IH Stroke (*n* = 542)	IH Stroke (*n* = 14)	*p* (IH)	No ML Stroke (*n* = 540)	ML Stroke (*n* = 16)	*p* (ML)
**Baseline Echocardiographic Parameters**							
LVEF (%)	55.0 (45.0–65.0)	55.0 (45.0–65.0)	52.5 (30.0–65.0)	0.529	55.0 (44.0–65.0)	60.0 (55.0–65.0)	0.086
Left atrial diameter (cm)	4.6 (4.3–5.0)	4.6 (4.3–5.0)	4.6 (4.5–4.9)	0.586	4.6 (4.3–5.0)	4.6 (4.2–5.0)	0.738
Aortic valve area (cm^2^)	0.7 (0.6–0.8)	0.7 (0.6–0.8)	0.7 (0.6–0.8)	0.585	0.7 (0.6–0.8)	0.7 (0.5–0.9)	0.967
Indexed AVA (cm^2^/m^2^)	0.4 (0.3–0.4)	0.4 (0.3–0.4)	0.3 (0.3–0.4)	0.230	0.4 (0.3–0.4)	0.4 (0.3–0.5)	0.322
Peak aortic jet velocity (m/s)	4.4 (4.1–4.8)	4.4 (4.1–4.8)	4.4 (4.0–4.7)	0.761	4.4 (4.1–4.8)	4.6 (4.1–5.3)	0.291
Peak transvalvular gradient (mmHg)	78.5 (67.3–93.0)	78.5 (68.0–93.0)	78.0 (64.0–90.0)	0.646	78.0 (67.0–93.0)	88.5 (69.3–109.0)	0.167
Mean transvalvular gradient (mmHg)	46.0 (41.0–58.0)	46.0 (41.0–58.0)	45.0 (40.0–52.0)	0.370	46.0 (41.0–58.0)	58.5 (42.0–72.8)	0.116
Systolic PA pressure (mmHg)	40.0 (35.0–55.0)	40.0 (34.8–55.0)	43.0 (37.5–61.3)	0.562	40.0 (35.0–55.0)	37.5 (30.0–58.8)	0.703
Mitral regurgitation grade, *n* (%)				**0.039**			0.861
None/Trace	129 (23.2)	129 (23.8)	0 (0.0)		124 (23.0)	5 (31.3)	
Mild (Grade 1)	241 (43.3)	236 (43.5)	5 (35.7)		235 (43.5)	6 (37.5)	
Moderate (Grade 2)	117 (21.0)	112 (20.7)	5 (35.7)		113 (20.9)	4 (25.0)	
Moderate–severe/Severe (3–4)	69 (12.4)	65 (12.0)	4 (28.6)		68 (12.6)	1 (6.3)	
Mitral stenosis, *n* (%)	69 (12.4)	65 (12.0)	4 (28.6)	0.083	68 (12.6)	1 (6.3)	0.707
Aortic regurgitation grade, *n* (%)				0.596			0.369
None/Trace	157 (28.2)	154 (28.4)	3 (21.4)		151 (28.0)	5 (31.3)	
Mild (Grade 1)	277 (49.8)	271 (50.0)	6 (42.9)		271 (50.2)	6 (37.5)	
Moderate–Severe (Grades 2–4)	122 (21.9)	117 (21.6)	5 (35.7)		118 (21.9)	5 (31.3)	
TTE annulus diameter (cm)	2.1 (2.0–2.3)	2.1 (2.0–2.3)	2.3 (2.1–2.4)	0.141	2.1 (2.0–2.3)	2.1 (2.0–2.4)	0.869
E-wave deceleration index	1.2 (1.2–1.3)	1.2 (1.2–1.3)	1.2 (1.2–1.3)	0.288	1.2 (1.2–1.3)	1.2 (1.2–1.3)	0.946
AS haemodynamic pattern, *n* (%)				0.635			0.123
High-gradient	380 (68.3)	368 (67.9)	12 (85.7)		372 (68.9)	8 (50.0)	
Low-flow low-gradient (LFLG)	35 (6.3)	35 (6.5)	0 (0.0)		35 (6.5)	0 (0.0)	
Paradoxical LFLG	7 (1.3)	7 (1.3)	0 (0.0)		7 (1.3)	0 (0.0)	
Very severe AS (VSAS)	134 (24.1)	132 (24.4)	2 (14.3)		126 (23.3)	8 (50.0)	
**Cardiac CT Parameters**							
CT annulus diameter 1 (mm)	27.0 (26.0–28.0)	27.0 (26.0–28.0)	26.0 (25.0–28.3)	0.332	27.0 (26.0–28.0)	26.5 (25.3–28.0)	0.250
CT annulus diameter 2 (mm)	22.0 (21.0–23.0)	22.0 (21.0–23.0)	21.5 (20.8–23.3)	0.687	22.0 (21.0–23.0)	21.5 (20.3–23.0)	0.418
Mean CT annulus diameter (mm)	24.5 (23.5–25.5)	24.5 (23.5–25.5)	23.8 (22.9–26.0)	0.424	24.5 (23.5–25.5)	23.8 (23.0–25.4)	0.238
CT annulus radius (mm)	12.2 (11.8–12.8)	12.3 (11.8–12.8)	11.9 (11.4–13.0)	0.424	12.3 (11.8–12.8)	11.9 (11.5–12.7)	0.238
CT annulus area (mm^2^)	471.2 (433.5–510.4)	471.2 (433.5–510.4)	442.8 (410.8–530.7)	0.424	471.2 (433.5–510.4)	442.8 (415.3–490.6)	0.202
CT annulus perimeter (mm)	76.9 (73.8–80.1)	76.9 (73.8–80.1)	74.6 (71.8–81.6)	0.424	76.9 (73.8–80.1)	74.6 (72.2–78.5)	0.202
Sinus of Valsalva diameter 1 (mm)	31.0 (29.0–33.0)	31.0 (29.0–33.0)	31.0 (28.8–34.0)	0.973	31.0 (29.0–33.0)	31.0 (28.0–32.8)	0.509
Sinus of Valsalva diameter 2 (mm)	31.0 (29.0–33.0)	31.0 (29.0–33.0)	33.0 (29.8–34.0)	0.215	31.0 (29.0–33.0)	30.5 (28.3–32.0)	0.319
Mean sinus of Valsalva diameter (mm)	31.0 (29.4–33.5)	31.0 (29.0–33.5)	32.3 (29.4–34.5)	0.487	31.0 (29.5–33.5)	30.8 (28.5–32.3)	0.355
Mean ascending aorta diameter (mm)	36.0 (34.0–38.5)	36.0 (34.0–38.5)	35.3 (33.4–38.8)	0.629	36.0 (34.0–38.5)	36.0 (33.0–37.9)	0.631
Aortic valve calcium score (Agatston) *	2860 (1275–5039)	2860 (1275–5039)	N/A	—	2860 (1275–5039)	N/A	—
Bicuspid aortic valve, *n* (%)	71 (12.8)	69 (12.7)	2 (14.3)	0.696	69 (12.8)	2 (12.5)	>0.999

Data are presented as median (interquartile range, IQR) for continuous variables, and as number (*n*) and percentage (%) for categorical variables. Between-group comparisons were performed using the Mann–Whitney U test for continuous variables and Fisher’s exact test or the chi-square test for categorical variables. Bold *p*-values denote statistical significance (*p* < 0.05). * Aortic valve calcium score was available in 15 patients only; no valid cases were present in either stroke group, so between-group testing was not performed. Abbreviations: IQR, interquartile range; LVEF, left ventricular ejection fraction; AVA, aortic valve area; PA, pulmonary artery; TTE, transthoracic echocardiography; AS, aortic stenosis; LFLG, low-flow low-gradient; VSAS, very severe aortic stenosis; CT, computed tomography; IH, in-hospital; ML, mid-to-long term, N/A, not applicable.

**Table 3:** Procedural Characteristics and Antithrombotic Therapy—Overall Cohort and by Stroke Status.

Variable	Overall (*n* = 556)	No IH Stroke (*n* = 542)	IH Stroke (*n* = 14)	*p* (IH)	No ML Stroke (*n* = 540)	ML Stroke (*n* = 16)	*p* (ML)
**Antithrombotic Therapy**							
Pre-procedural aspirin, *n* (%)	405 (72.8)	398 (73.4)	7 (50.0)	0.162	393 (72.8)	12 (75.0)	0.813
Clopidogrel, *n* (%)	3 (0.5)	3 (0.6)	0 (0.0)		3 (0.6)	0 (0.0)	
Pre-procedural warfarin, *n* (%)	110 (19.8)	105 (19.4)	5 (35.7)		107 (19.8)	3 (18.8)	
Novel oral anticoagulant, *n* (%)	19 (3.4)	18 (3.3)	1 (7.1)		18 (3.3)	1 (6.3)	
Aspirin + Clopidogrel, *n* (%)	19 (3.4)	18 (3.3)	1 (7.1)		19 (3.5)	0 (0.0)	
**Procedural Characteristics**							
Transfemoral closure technique, *n* (%)				0.270			0.851
Prostar	179 (32.2)	174 (32.1)	5 (35.7)		173 (32.0)	6 (37.5)	
Proglide	365 (65.6)	357 (65.9)	8 (57.1)		355 (65.7)	10 (62.5)	
Cut-down	12 (2.2)	11 (2.0)	1 (7.1)		12 (2.2)	0 (0.0)	
Pre-dilatation (BAV), *n* (%)	400 (71.9)	388 (71.6)	12 (85.7)	0.369	386 (71.5)	14 (87.5)	0.257
Post-dilatation, *n* (%)	19 (3.4)	18 (3.3)	1 (7.1)	0.389	16 (3.0)	3 (18.8)	0.014
Prosthesis size (mm), *n* (%)				0.928			0.595
20 mm	2 (0.4)	2 (0.4)	0 (0.0)		2 (0.4)	0 (0.0)	
23 mm	232 (41.7)	226 (41.7)	6 (42.9)		226 (41.9)	6 (37.5)	
25 mm	14 (2.5)	14 (2.6)	0 (0.0)		13 (2.4)	1 (6.3)	
26 mm	229 (41.2)	222 (41.0)	7 (50.0)		221 (40.9)	8 (50.0)	
27 mm	6 (1.1)	6 (1.1)	0 (0.0)		6 (1.1)	0 (0.0)	
29 mm	73 (13.1)	72 (13.3)	1 (7.1)		72 (13.3)	1 (6.3)	
Valve-in-valve procedure, *n* (%)	7 (1.3)	7 (1.3)	0 (0.0)	>0.999	7 (1.3)	0 (0.0)	>0.999
Device success (VARC-3), *n* (%)	535 (96.2)	521 (96.1)	14 (100.0)	>0.999	519 (96.1)	16 (100.0)	0.654

Data are presented as number (*n*) and percentage (%) for categorical variables. Between-group comparisons were performed using Fisher’s exact test or the chi-square test; bold *p*-values denote statistical significance (*p* < 0.05). Device success was defined according to Valve Academic Research Consortium-3 (VARC-3) criteria. Abbreviations: BAV, balloon aortic valvuloplasty; VARC-3, Valve Academic Research Consortium-3; IH, in-hospital; ML, mid-to-long term.

**Table 4:** Clinical Outcomes and Follow-Up—Overall Cohort.

Variable	Overall Cohort (*n* = 556)
**Prosthesis Type**	
SAPIEN XT, *n* (%)	479 (86.2)
SAPIEN 3, *n* (%)	46 (8.3)
Lotus, *n* (%)	24 (4.3)
Other valve types, *n* (%)	7 (1.3)
**In-Hospital Events**	
In-hospital stroke, *n* (%)	14 (2.5)
Pericardial tamponade, *n* (%)	10 (1.8)
New-onset arrhythmia—AV complete block, *n* (%)	38 (6.8)
New-onset arrhythmia—Ventricular tachycardia, *n* (%)	3 (0.5)
New-onset arrhythmia—Atrial fibrillation, *n* (%)	20 (3.6)
New-onset arrhythmia—Left bundle branch block, *n* (%)	14 (2.5)
Permanent pacemaker implantation, *n* (%)	40 (7.2)
Peripheral vascular complications, *n* (%)	37 (6.7)
Haematoma, *n* (%)	5 (0.9)
Pseudoaneurysm, *n* (%)	9 (1.6)
Dissection, *n* (%)	19 (3.4)
Haemorrhage, *n* (%)	4 (0.7)
Other complications, *n* (%)	22 (4.0)
Valve embolisation, *n* (%)	4 (0.7)
Coronary obstruction, *n* (%)	2 (0.4)
Acute kidney injury, *n* (%)	4 (0.7)
Closure device failure, *n* (%)	11 (2.0)
Annular rupture, *n* (%)	1 (0.2)
In-hospital mortality, *n* (%)	22 (4.0)
**Post-TAVI Echocardiographic Outcomes**	
Post-procedural LVEF (%), median (IQR)	60.0 (45.8–65.0)
Post-procedural peak transvalvular gradient (mmHg), median (IQR)	19.0 (16.0–23.0)
Post-procedural mean transvalvular gradient (mmHg), median (IQR)	10.0 (8.0–12.0)
Post-procedural paravalvular AR—Mild, *n* (%)	531 (95.5)
Post-procedural paravalvular AR—Moderate, *n* (%)	25 (4.5)
Time from procedure to last echocardiography (months), median (IQR)	24.0 (15.0–33.0)
Follow-up NT-proBNP (pg/mL), median (IQR) *	1594 (637–2956)
**Mid-to-Long Term Follow-Up**	
30-day mortality, *n* (%)	33 (5.9)
6-month mortality, *n* (%)	40 (7.2)
1-year mortality, *n* (%)	91 (16.4)
Mid-to-long term stroke, *n* (%)	16 (2.9)

Data are presented as number (*n*) and percentage (%) for categorical variables, and as median (interquartile range, IQR) for continuous variables. Clinical outcomes are reported for the overall cohort. Device success and procedural definitions followed Valve Academic Research Consortium-3 (VARC-3) criteria. Post-procedural echocardiographic data reflect the last available study following the index procedure. Follow-up NT-proBNP was available in 31 patients; * indicates that follow-up NT-proBNP data were available in 31 patients. Abbreviations: IQR, interquartile range; AV, atrioventricular; LVEF, left ventricular ejection fraction; NT-proBNP, N-terminal pro-B-type natriuretic peptide; TAVI, transcatheter aortic valve implantation.

**Table 5:** Firth Penalized Multivariable Logistic Regression for Predictors of Stroke.

Variable	β	SE	Adjusted OR	95% CI	*p*-Value
**In-hospital stroke**					
Baseline mitral regurgitation grade	0.777	0.263	2.174	1.299–3.638	0.003
HbA1c (%)	0.497	0.200	1.644	1.111–2.433	0.013
Prior cerebrovascular event	1.451	0.669	4.269	1.150–15.846	0.030
**Mid-to-long term stroke**					
Post-dilatation	2.289	0.689	9.862	2.555–38.067	<0.001
Very severe aortic stenosis phenotype	1.294	0.510	3.649	1.343–9.916	0.011

Firth’s penalized-likelihood logistic regression was used in both models to address sparse-data bias arising from the low number of events. Only variables retained by the forward stepwise selection are shown. In-hospital stroke, 14 events: the full candidate set entered into the model comprised age, STS score, prior cerebrovascular event, atrial fibrillation, obstructive coronary artery disease, pre-procedural aspirin, HbA1c, baseline mitral regurgitation grade, and mitral stenosis. Baseline mitral regurgitation grade was modelled as an ordinal variable, and its odds ratio reflects the change in odds per one-grade increase. Mid-to-long term stroke, 16 events: the full candidate set entered into the model comprised age, prior cerebrovascular event, atrial fibrillation, post-dilatation, very severe aortic stenosis phenotype, and baseline mean transvalvular gradient. Abbreviations: OR, odds ratio; CI, confidence interval; SE, standard error; HbA1c, glycated haemoglobin.

## Data Availability

The datasets generated and/or analyzed during the current study are not publicly available due to institutional and privacy restrictions but are available from the corresponding author on reasonable request.
